# Psychometric evaluation of the academic involution scale for college students in China: An application of Rasch analysis

**DOI:** 10.3389/fpsyg.2023.1135658

**Published:** 2023-02-21

**Authors:** Yanchao Yang, Yan Peng, Wangze Li, Shan Lu, Chen Wang, Sirui Chen, Jialiang Zhong

**Affiliations:** ^1^North China University of Science and Technology, Tangshan, China; ^2^Faculty of Education, University of Macau, Taipa, Macao SAR, China; ^3^Lyceum of the Philippines University-Batangas, Batangas, Philippines; ^4^Sehan University, Yeongam County, Republic of Korea; ^5^Pukyong National University, Busan, Republic of Korea

**Keywords:** academic involution, Rasch analysis, scale development, scale validation, college students

## Abstract

Recent academic attention on educational involution in China underpins the need for a valid and reliable instrument to precisely measure college students’ academic involution behaviors. Seeing the scarcity of a proper instrument, the current study attempted to analyze the item-level psychometric properties of the newly developed Academic Involution Scale for College Students (AISCS) in China by using a Rasch model. A total of 637 college students in a public university in northern China participated in the study. Data were examined with respect to unidimensionality, rating scale functioning, item fit statistics, item polarity, item- and person-level reliability and separation, item hierarchy and invariance across educational background with Winsteps. The results show that AISCS was a single unidimensional construct with good psychometric properties. Although two items demonstrated differential item functioning, it is plausible given the differences between assessment methods for undergraduates and postgraduates. Limitations and future research directions with regard to sample selection, inclusion of more validity evidence and adding prospective additional academic involution were discussed.

## 1. Introduction

The concept of involution was originally coined by Geertz in the agricultural industry and it was defined as cultural patterns that do not stabilize or improve toward a new pattern and continue to evolve by becoming more internally complex after reaching a seemingly definitive form Geertz (1963). Now, the term “involution” has been widely used in diverse fields across agriculture, politics, economics, education and so on (Li, 2021). What’s more, a close look at the literature on involution indicated that the concept has been mainly researched in Asian countries, such as in Japan (Mihara, 2020), and China (Li, 2021; Xue, 2021; Cai, 2022; Chen et al., 2022; Li, 2022; Si, 2022; Yu et al., 2022). With the research focus of Mihara (2020) on Japanese animation industry, the majority of prior studies on involution in China concentrate on education. Currently, in a higher education setting, educational involution or academic involution denotes a type of behavior that shows increasing negativity, excessive competition, and low productivity among college students in an irrationally competitive environment (Zheng et al., 2022).

Prior studies on educational involution in China mainly focus on the theoretical rationales (Yi et al., 2022), the features (Guo, 2022), reasons and the detrimental effects (Li, 2021) as well as its relationship with other variables (Yan et al., 2022). What’s more, the target participants for educational involution research varies from the perspectives of parents (Yu et al., 2022), students (Liu et al., 2022; Yan et al., 2022; Yi et al., 2022) and young academics (Si, 2022). Specifically, Yi et al. (2022) attempted to analyze the rationale for educational involution from the perspectives of Classical Social Comparison Theory, Achievement Motivation Theory, and Cognitive Evaluation Theory. They proposed that when facing peer pressure or extrinsic motivation, students tended to engage themselves in educational involution either in an active or submissive manner for limited social resources. Furthermore, educational involution is characterized by instrumentalist academic situations, overly competitive interpersonal relationships, anxious psychological states, and limited employment opportunities (Guo, 2022). In addition, the reasons for educational involution were listed as unequal distribution of education resources and the benefits of education in Chinese society (Li, 2021). The influences were cited as homogeneity in society, entrenched education inequity and self-flagellation (Li, 2021). Furthermore, as for the relationship with other variable, the results of the study by Yan et al. (2022) showed that there was a significant and positive correlation between educational involution behaviors and anxiety as well as stress.

However, a close look at the measurement of educational involution reveals the scarcity of reliable and valid instrument. Although an instrument specifically measuring academic involution behavior was proposes by Yan et al. (2022), it did not to provide the validity evidence. In addition, the College Student Involution Behavior Scale (Yi et al., 2022) attempted to cover students’ involution behaviors from three theoretical aspects, but failed to include social relations with their classmates and roommates as one element given the important role that peer appraisal plays in scholarship assessment. To present valid and reliable results for policies, scales with sound psychometric properties are required (Andrich, 1988). Therefore, the current study attempted to examine the item-level psychometric properties of the newly designed academic involution scale in the Chinese higher education context. Specifical research questions (RQ) were proposed as follows:

- RQ1: Does the academic involution scale exhibit item-level psychometric properties to effectively assess Chinese college students’ academic involution behaviors as a single unidimensional construct?
- RQ 2: Does the five-point Likert scale function appropriately?
- RQ 3: Do the 16 items demonstrate acceptable item fit statistics?
- RQ 4: Is the item difficulty hierarchy consistent with our expectation?
- RQ 5: Are the person- and item-level separation and reliability acceptable?
- RQ 6: Does the scale measure invariantly across demographic factors, such as educational background (undergraduates versus postgraduates)?

## 2. Methods

### 2.1. Participants

A total of 637 college students in a provincial-level key university in Hebei, China participated in the current study. Convenience sampling was adopted because of most of the authors have been working in that university for more than 6 years. To reduce social desirability bias, the respondents remained anonymous during the data collection, and only educational background information was obtained. Most of participants (84%) were undergraduates. Detailed information was shown in Table 1.

**Table 1:** General information of the participants.

		Frequency	Percent	Valid percent	Cumulative percent
Valid	Undergraduate	535	84.0	84.0	84.0
	Postgraduate	102	16.0	16.0	100.0
	Total	637	100.0	100.0	

### 2.2. Instrument

The 16-item questionnaire, AISCS, was developed to measure college students’ involution behaviors. The scale is designed to measure the following three aspects: (1) learning (Items L1 to L7, for instance, *I will attend a tutorial class privately to improve myself so as not to be left behind by others*), (2) activity (Items A1to A5, for instance, *Although I do not like it very much, I will attend various lectures so that my comprehensive evaluation results will not be left behind by others.*) and (3) social relations (Items, SR1- SR4, for instance, *I will actively interact with teachers and strive to achieve no lower grades than others*). The three aspects were summarized based on interviews with college students. The items were generated informed by the 2 features of academic involution of increasing negativity, excessive competition (Zheng et al., 2022). For instance, phrases such as “so as to avoid being left behind by others,” “in order to get better results” reflected the nature of “excessive competition.” In addition, expression such as “Although I do not like it very much” corresponded to the nature of “increasing negativity.” All 16 items were measured on a 5-point rating scale from 1 (Strongly disagree) to 5 (Strongly agree). It was administered to the participants with the help of the third author *via* Wenjuanxing, which is a widely used online questionnaire website. Before the administration, ethical approval was granted and consent was sought from the college students.

### 2.3. Data analytical procedure

Respondents need to distinguish each category when responding to a Likert scale with several categories. In the current study, participants need to carefully interpret the distance between neighboring category, for instance between 5 (totally disagree) and 4 (disagree), and so forth. If this distance measured in logits between neighboring categories varies across the items, this corresponds to a Partial Credit Model (Wright and Masters, 1982). However, if the distance measured in logits between neighboring categories across items is interpreted as equivalent, this corresponds to a Rating Scale Model (Andrich, 1978; Bond and Fox, 2015). In the current study, all 16 items share the same response pattern, therefore, after data were gathered, Rasch Rating Scale Model with Winsteps software (Linacre, 2012) was adopted.

Next, six aspects of Rasch model fit statistics were examined, specially, unidimensionality, rating scale functioning (monotonicity), item fit with infit and outfit MNSQ and item polarity, item- and person-level reliability and separation, targeting examined by Wright Map and the difference between mean of the person ability and that of the item endorseability, and differential item functioning (DIF). The six aspects and their criteria were explained in the following analytical procedure.

First one of the basic assumptions of the Rasch model, unidimensionality, was examined. Unidimensionality refers to the existence of a primary construct that explains the variance in sample response. The variance explained by the primary construct corresponds to the Rasch dimension, while the unexplained variance refers to all other dimensions and random noise. In the current study, principal component analysis of residuals (PCAR) was used to examine the unidimensionality (Smith, 2002). PCAR attempts to partition the unexplained variance based on factors representing other dimensions (Linacre, 2006). The following criteria were adopted (1) Rasch dimension should explain the variance by at least 40% (Linacre, 2006), (2) the first contrast (the largest secondary dimension after the Rasch dimension is removed) should account for the variance by <15%, (3) the unexplained variance of the eigenvalue for the first contrast should be <3.0 (Galli et al., 2008; Linacre, 2019) with a minimum ratio of 3:1 between the variance explained by the Rasch dimension and that by first contrast (Embretson and Reise, 2000).

Second, another assumption of Rasch model, monotonicity, was then investigated. The monotonicity assumption indicates that the probability of endorsing an item increase as the trait level increases. In other words, the probability of more extreme or greater responses on an item corresponds with a greater amount of the latent trait being measured. In the current study, it was analyzed with regards to the following criteria (Linacre, 2002; Bond and Fox, 2015): (1) there should be at least 10 cases per category (Smith et al., 2003), (2) the average measures increases monotonically across categories (Linacre, 2002), (3) the values for category outfit mean square (MNSQ) should be <2.0 (Bond and Fox, 2015), and (4) the difficulty of endorsement for rating scale categories increases by at least 1.4 logits but not more than 5 logits between categories (Linacre, 1999).

Third, Rasch item fit statistics demonstrate how well the data fit the Rasch measurement model (Linacre, 2002), which could be used to identify ‘mis-fitting’ items. In the current study, item statistics were investigated by examining the Infit and outfit Mean square fit statistics (MNSQ) and item polarity. Infit MNSQ, which is more sensitive to unexpected response of persons whose abilities are near item difficulty, is an information-weighted mean square residual, while outfit MNSQ, which is more sensitive to unexpected outlying observations, is unweighted mean square residual (Tennant and Conaghan, 2007). Generally, the recommended cut-off value for MNSQ is between 0.75 and 1.33 (Wilson, 2004). In addition, the item polarity, displayed by point measure correlation (PTMEA CORR) coefficient, was also examined. Items with high PTMEA CORR values are expected to distinguish respondents’ ability properly. The cut-off value between +0.4 logit and + 0.8 logit (0.4 < x < 0.8) was adopted (Fisher, 2007; Linacre, 2012).

Fourth, person- and item-level reliability and separation were investigated. The Rasch person reliability index indicates the replicability of subject ordering if the sample of persons was given another set of items that measured the same construct and the item reliability index indicates the internal consistency reliability of multi-item scales. Both of these reliability estimates have a threshold of 0.70 to be regarded as acceptable, of 0.80 to be considered satisfactory and of 0.90 deemed as excellent (Bond and Fox, 2015). The person separation can be used to identify the number of statistically distinct ability strata of the individuals in the sample (Bond and Fox, 2015), and item separation is used to verify the item hierarchy. The cut-off value for person separation is above 2 and for item separation is at least 4 (Malec et al., 2007).

Fifth, item hierarchy was further examined by referring to the Wright map, which displays person ability and item endorsability measured on the same logit scale. Wright map was adopted to identify whether the endorsability of instrument items is consistent with person abilities. In order for the items of the instrument to precisely measure person abilities, the endorsability of the instrument should accurately match person ability (Boone, 2016). A difference of more than 1 logit between item endorsability and person ability indicates mistargeting (Mallinson et al., 2004; Pesudovs et al., 2007; McAlinden et al., 2012; Planinic et al., 2019; Cantó-Cerdán et al., 2021).

Finally, the generalizability aspect of scale validity was inspected *via* testing the differential item functioning (DIF) of items across demographic variables. Rasch model requires that subgroups of participants who share equivalent levels of the underlying construct, should respond similarly to the items that measure that construct (Tennant and Conaghan, 2007). The existence of DIF negatively impacts the quality of measure instrument. In the current study, a conservative cut-off DIF contrast value of ≥0.5 logit difference was used (Wang, 2008). If the items are identified as DIF-biased across different groups, some measures can be recommended such as deleting items, adding new ones, or developing separate measures for specific subgroups. In the current study, DIF was examined in reference to the participants’ educational background (undergraduate versus postgraduate) since other demographic information was not available.

## 3. Results

### 3.1. Unidimensionality

PCAR results in Table 2 revealed that 47.3% of total variance was explained by the Rasch dimension, exceeding the recommended 40% (Linacre, 2006). In addition, the variance explained by the first contrast is 12.8%, less than the recommended cut-off value, 15%. What’s more, the unexplained variance of the eigenvalue for the first contrast is 3.9, a slightly larger than the recommended cut-off value, 3.0 (Galli et al., 2008; Linacre, 2019). However, the ratio between variance in the measurement dimension compared to the variance of the first contrast is about 3.7 (47.3%/12.8% = 3.7), larger than the 3:1 ratio recommend by Embretson and Reise (2000). Therefore, the results indicated that the AISCS fits the Rasch model, offering some statistical evidence of a unidimensionality measurement of AISCS.

**Table 2:** Standardized residual variance (in eigenvalue units).

		Empirical			Modeled
Total raw variance in observations	=	30.4	100.00%		100.00%
Raw variance explained by measures	=	14.4	47.30%		47.20%
Raw variance explained by persons	=	7.7	25.40%		25.30%
Raw Variance explained by items	=	6.7	22.00%		21.90%
Raw unexplained variance (total)	=	16.0	52.70%	100.00%	52.80%
Unexplained variance in 1st contrast	=	3.9	12.80%	24.30%	
Unexplained variance in 2nd contrast	=	3.0	9.90%	18.90%	
Unexplained variance in 3rd contrast	=	1.2	4.10%	7.80%	
Unexplained variance in 4th contrast	=	1.1	3.70%	7.10%	
Unexplained variance in 5th contrast	=	1.0	3.40%	6.40%	

### 3.2. Rating scale functioning

**Figure 1:**
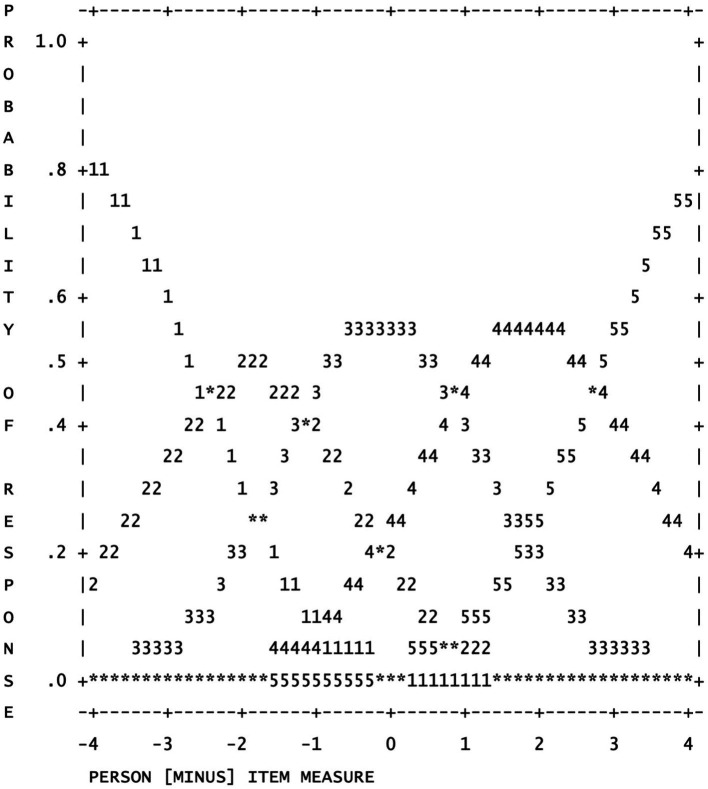
Category probabilities: Modes - structure measures at intersections.

The results in Table 3 indicated that the 5-point AISCS functioned well. The observed count ranged from 618 to 4,143, exceeding the recommended cut-off value 10 for each rating category and the Outfit MNSQ (ranging from 0.82 to 1.28) were less than recommended cut-off value 2, displaying adequate fit (Linacre, 2002). What’s more, the average measure ranging from −2.17 to 1.70 increased with the category level. Furthermore, the category thresholds advanced with the category level. In addition, the shape of each rating scale distribution as shown in Figure 1 also provided that the scale functioned well. For instance, each category had a distinct pick, suggesting that participants reliably distinguished response categories.

**Table 3:** Summary of rating scale function.

Category	Observed count	Average measure	Infit MNSQ	Outfit MNSQ	Category thresholds
1	920	−2.17	1.08	1.12	NONE
2	2,163	−0.99	0.85	0.82	−2.46
3	4,143	−0.06	0.87	0.90	−1.15
4	2,348	0.74	1.00	1.02	0.86
5	618	1.70	1.30	1.28	2.75

### 3.3. Item fit and item polarity

The item statistics displayed in Table 4 indicated no misfitting items. The infit MNSQ values, ranging from 0.77 to 1.34, and outfit MNSQ values, ranging from 0.77 to 1.32 displayed that the data adequately fitted to the Rasch model. Furthermore, the PTMEA CORR coefficient in Table 3 was more than 0.38, indicating the 16 items contributed to the measurement of academic involution behaviors.

**Table 4:** Item statistics: misfit order.

Item	Measure	INFIT MNSQ	OUTFIT MNSQ	PTMEA CORR
L2	−0.25	1.34	1.32	0.61
L5	−0.29	1.24	1.30	0.64
L3	−0.43	1.24	1.25	0.61
L4	−0.01	1.16	1.17	0.64
L1	1.46	1.13	1.12	0.60
L6	−0.29	1.07	1.05	0.65
SR3	−0.38	0.95	0.97	0.66
SR1	−0.55	0.93	0.97	0.65
A2	0.3	0.93	0.94	0.72
L7	0.37	0.92	0.93	0.67
A3	0.10	0.92	0.91	0.73
SR4	−0.43	0.89	0.88	0.68
A1	0.20	0.85	0.89	0.73
A5	0.18	0.83	0.81	0.75
SR2	−0.35	0.79	0.78	0.71
A4	0.34	0.77	0.77	0.75

### 3.4. Reliability and separation

The results in Table 5 indicated that AISCS was highly reliable with Rasch item reliability of 0.99 and person reliability of.90. The item separation of 8.32 and person separation of 3.05 were also satisfactory. The index of item separation of 4 or greater and the index of person separation of 2 or greater are desirable (Malec et al., 2007). Therefore, the results indicated that AISCS was sensitive to differences between respondents with varying levels of involution for college students.

**Table 5:** Item- and person-level reliability and separation.

	Item	Person
Separation	8.32	3.05
Reliability	0.99	0.90

### 3.5. Targeting

**Figure 2:**
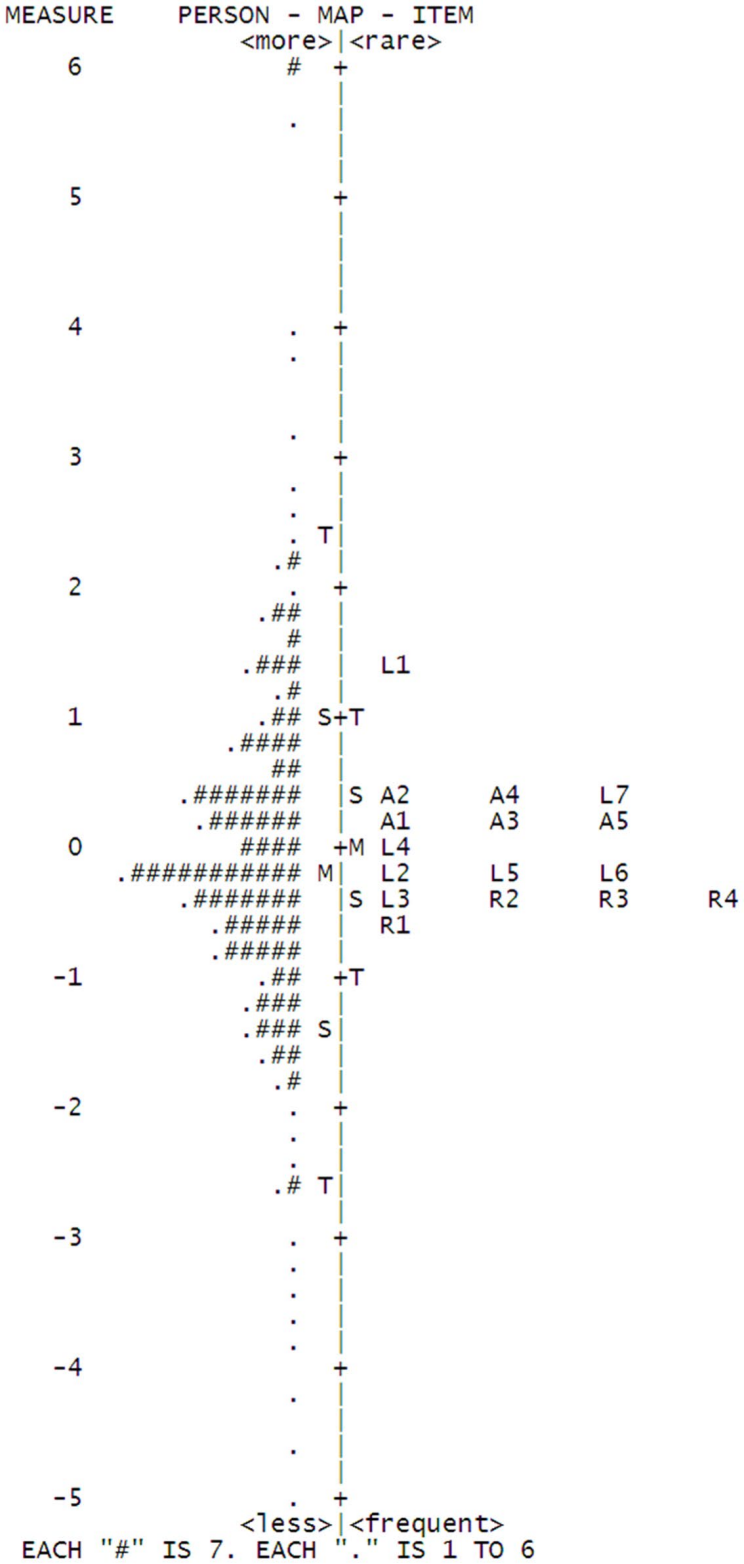
Wright map.

The results indicated the items were well targeted. The item endorsability ranged from −0.55 to +1.46 logits. Overall, the levels of item endorsability matched well with our expectations that involution in learning aspects is more common for college students. As shown in Wright map in Figure 2, L1 was estimated to be the most difficult item for students, followed by L7, both of which are Learning-related. R1 was estimated to be the easiest one since dorm-mates play a less decisive role for students’ involution. It is plausible in some cases students from different classes or majors will live together and some students choose to live alone. Therefore, it will be less likely for students to endorse this item to represent involution.

In addition, the mean of the person ability was very close to that of the item endorseability with a logit difference of.14. The mean for both respondent ability and item endorseability are approximately around the same location, thus indicating that the items for this sample are well targeted.

### 3.6. DIF

We also analyzed DIF to identify potential item bias across particular groups of participants. Specifically, we would like to ensure that items are invariant for both undergraduates and postgraduate students. However, the results in Table 6 indicated that of the 16 items, 2 items (L3 *In order to get better results, I will consult with the senior students about the relevant knowledge of the courses I have registered such as the past exam questions, test materials, and teacher’s notes* and L4 *in order to achieve excellent results on the final exam, I will purchase some learning resources such as PPT templates, reference books, past exam questions*, etc.) were extracted as biased items that showed statistically significant DIF based on educational background. The two items are both related to expectation in final examination. For undergraduates, examination is a major assessment tool. For postgraduates, assessments will be conducted in a more diverse manner, such as presentation and thesis writing. The two items are therefore expected to be considered bias in reference to educational background.

**Table 6:** DIF across educational background.

	Undergraduates			Postgraduates			
Item	Observations average	DIF measure	DIF S.E.	Observations average	DIF measure	DIF S.E.	DIF contrast
L1	−0.03	1.51	0.06	0.14	1.17	0.14	0.35
L2	−0.03	−0.19	0.06	0.17	−0.57	0.14	0.38
L3	−0.05	−0.32	0.06	0.30	−1.02	0.14	**0.69**
L4	−0.05	0.09	0.06	0.28	−0.55	0.14	**0.64**
L5	0.01	−0.29	0.06	−0.05	−0.19	0.14	−0.09
L6	0.00	−0.29	0.06	−0.01	−0.29	0.14	0.00
L7	−0.03	0.43	0.06	0.17	0.05	0.14	0.39
A1	0.02	0.17	0.06	−0.09	0.38	0.14	−0.21
A2	0.01	0.28	0.06	−0.08	0.46	0.14	−0.19
A3	0.04	0.03	0.06	−0.19	0.48	0.14	−0.45
A4	0.00	0.34	0.06	−0.02	0.38	0.14	−0.04
A5	0.02	0.15	0.06	−0.11	0.4	0.14	−0.26
SR1	0.03	−0.61	0.06	−0.17	−0.21	0.14	−0.4
SR2	0.02	−0.38	0.06	−0.08	−0.19	0.14	−0.19
SR3	0.01	−0.4	0.06	−0.07	−0.25	0.14	−0.15
SR4	0.03	−0.49	0.06	−0.18	−0.07	0.14	−0.42

## 4. Discussion

The results of the current study supported the unidimensionality of AISCS, indicating that a single underlying construct, i.e., academic involution could account for the majority of the variance. Therefore, the results provided evidence for the construct validity for the scale. What’s more, regarding the rating scale properties, the recommend 4 criteria proposed by Linacre (2002) for rating scale functioning were all satisfied. There were enough observed frequencies for each category, the average measure increased monotonically along the categories, outfit for all categories were well below 2.0 and close to 1.0, and the thresholds also increased monotonically indicating that each category is the most probable for a specific range on the construct continuum. Furthermore, the psychometric results of the scale were satisfactory with no misfitting item and high reliability and separation for both item and person. In other words, the current AISCS had a capability to differentiate between different levels of responding person sample (Linacre, 2019). In addition, the item-person person map indicated the items for this sample were well targeted. Finally, the 16-item AISCS was examined for DIF based on educational background (undergraduates versus postgraduates). It was found that 2 items related to examination exhibited DIF, which is consistent with our expectation.

## 5. Implications

A reliable and valid instrument can help stakeholders accurately measure specific construct, because reliability and validity are the prerequisites of an instrument to guarantee the subsequent measurement integrity and quality. The AISCS in the current study verified through Rasch analysis may assist teachers to identify students’ academic involution behaviors and help stakeholders adopt precautionary strategies to prevent students from engaging in irrationally and excessively competitive activities.

In addition, a reliable and valid instrument on academic involution behaviors for college students can help researchers conduct quantitative research to identify antecedents and consequences through path analysis. This is especially of significance for Chinese students who face the fiercest academic competition and shrinking employment opportunities.

## 6. Limitation and future direction

There are a few potential limitations to this study. First, the study sample was comprised only of college students conveniently selected from a public college in northern China. It is also noteworthy that given the role of researchers in the process of students’ scholarship assessment, participants may tend to respond in ways according to how they think their responses will be viewed by others, instead of answering truthfully. In future studies, participants, hopefully from other universities, should be included to reduce social desirability bias.

Second, in the current study, to reduce social desirability bias, only one kind of demographic data (educational background) was collected. Therefore, DIF was merely examined across educational background. To examine whether scale is invariant across other demographic variables, future studies are suggested to include more diverse demographic variables, such as gender, grade level, school level, school location and school type.

Third, the current study provided several aspects of validity by using Rasch analysis, such as structural validity (Rasch dimensionality analyses), generalizability (differential item functioning and person separation reliability), and substantive validity (rating scale functioning, and item difficulty hierarchy). More aspects of validity such as evidence of external validity should be conducted in future studies using Classical Test Theory by correlating the responses to other theoretically related variables with response collected by the current scale.

Fourth, the items were generated based two features of involution, increasing negativity, excessive competition (Zheng et al., 2022). Another feature, low productivity was not covered. One limitation of the scale could be less attention to the fact that some students tended to engage themselves in educational involution in either an active manner or submissive manner (Yi et al., 2022). Low productivity may apply to students who engage in involution in a submissive manner. Therefore, in future studies, items or dimensions focusing on low productivity should be added to provide a holistic picture of students’ academic involution behaviors.

Fifth, although the results of the current study in relation to monotonicity meet the minimal criteria for model fit based on Rasch Rating Scale Model, the observed counts for extreme options (strongly agree and strongly disagree) were comparatively lower than that for other three options. This might be explained by the homogeneity of the participants most of whom are facing the moderate level of academic pressure in a provincial-level university. Future studies may involve participants from universities of different levels in one study, for instance, inviting participants from municipal-level colleges, provincial-level universities and national-level universities. In addition, other Rasch models may be adopted to provide more robust and defensible results, such as Partial Credit Model, which also applies to Liker-type questionnaires whose response categories are adequately not observed.

Finally, considering the multiple aspects of the scale (learning, activities and social relations) and evidence for the unidimensionality supported by the PCAR results, future studies are suggested to test the structure of the scale by using Exploratory Factor Analysis and Confirmatory Factor Analysis, especially a second-order Confirmatory Factor Analysis to test whether the theorized academic involution in the current study loads into certain number of underlying sub-constructs or components. In addition, although the scale includes three aspects, more prospective aspects such as research and employment should be included to provide a more comprehensive picture about the educational involution for college students.

In summary, our findings provided support for the psychometric properties of the newly developed AISCS. Although further revision and validation of the scale is needed to determine its utility with more diverse samples, the scale fills a void for researchers and educators who need to assess educational involution, based on the strength of the results presented.

## Data availability statement

The raw data supporting the conclusions of this article will be made available by the authors, without undue reservation.

## Ethics statement

The studies involving human participants were reviewed and approved by North China University of Science and Technology. The patients/participants provided their written informed consent to participate in this study.

## Author contributions

YY conceived of the presented idea and completed the draft. WL helped collect data and provided insightful comments on implication section. YP, SL, CW, SC, and JZ contributed to the final version of the manuscript. All authors contributed to the article and approved the submitted version.

## Funding

This manuscript is funded by China Hebei Provincial Medical Science Research Key Project Fund Project (20210103) and China Hebei Provincial University Fundamental Scientific Research Operation Fund Project Science and Technology Basic Research Project (JQN2020011).

## Conflict of interest

The authors declare that the research was conducted in the absence of any commercial or financial relationships that could be construed as a potential conflict of interest.

## Publisher’s note

All claims expressed in this article are solely those of the authors and do not necessarily represent those of their affiliated organizations, or those of the publisher, the editors and the reviewers. Any product that may be evaluated in this article, or claim that may be made by its manufacturer, is not guaranteed or endorsed by the publisher.

## References

[ref1] Andrich D. (1978). Application of a psychometric rating model to ordered categories which are scored with successive integers. Appl. Psychol. Meas. 2, 581–594. doi: 10.1177/014662167800200413

[ref2] Andrich D. (1988). Rasch Models for Measurement, vol. 68. Newbury Park, CA: Sage.

[ref3] Bond T. Fox C. M. (2015). Applying the Rasch Model: Fundamental Measurement in the Human Sciences. Newbury Park, CA: Routledge.

[ref4] Boone W. J. (2016). Rasch analysis for instrument development: why, when, and how? CBE Life Sci. Educ. 15:rm4. doi: 10.1187/cbe.16-04-0148 27856555 PMC5132390

[ref5] Cai Y. (2022). The narrative symptoms and cultural context of “involution”-- taking college student group as an example. Adv. Educ. Hum. Soc. Sci. Res. 2, 320–326. doi: 10.56028/aehssr.2.1.320

[ref6] Cantó-Cerdán M. Cacho-Martínez P. Lara-Lacárcel F. García-Muñoz Á. (2021). Rasch analysis for development and reduction of symptom questionnaire for visual dysfunctions (SQVD). Sci. Rep. 11, 1–10. doi: 10.1038/s41598-021-94166-9 34290288 PMC8295373

[ref7] Chen Y. Han D. Liu Z. He W. Li X. (2022). The phenomena of “education involution” and the root cause cracking. Front. Bus. Econ. Manag. 6, 90–93. doi: 10.54097/fbem.v6i1.2288

[ref8] Embretson S. E. Reise S. P. (2000). “Item Response Theory for Psychologists” in Item response theory for psychologists. ed. Harlow Lisa L. (Mahwah: Lawrence Erlbaum Associates Publishers), xi, 371.

[ref9] Fisher W. P. (2007). Rating scale instrument quality criteria. Rasch Meas. Trans. 21:1095.

[ref10] Galli S. Chiesi F. Primi C. (2008). The construction of a scale to measure mathematical ability in psychology students: an application of the Rasch model. Test. Psicometria Metodol. 15, 1–16.

[ref11] Geertz C. (1963). Agricultural Involution: The Processes of Ecological Change in Indonesia, vol. 11. Los Angeles, California: Univ of California Press.

[ref12] Guo Y. L. (2022). Study on the representation and attribution of “introversion” of contemporary college students in China——from the perspective of discourse analysis. Meitan High. Educ 5, 65–71. doi: 10.16126/j.cnki.32-1365/g4.2021.05.009

[ref13] Li C. (2021). From Involution to Education: A Glance to Chinese Young Generation. 2021 4th International Conference on Humanities Education and Social Sciences (ICHESS 2021), 1884–1887.

[ref14] Li R. (2022). Analysis on the Chinese anxiety of involution from Jiwa with the background of globalization. Adv. Soc. Sci. Educ. Hum. Res. 615, 2491–2494.

[ref15] Linacre J. M. (1999). Investigating rating scale category utility. J. Outcome Meas. 3, 103–122. 10204322

[ref17] Linacre J. M. (2002). Understanding Rasch measurement: optimizing rating scale category effectiveness. J. Appl. Meas. 3, 85–106. 11997586

[ref18] Linacre J. M. (2006). Data variance explained by Rasch measures. Rasch Meas. Trans. 20:1045.

[ref19] Linacre John M. (2012). Winsteps® Rasch Measurement Computer Program User’s Guide. Beaverton, Oregon: Winsteps.Com.

[ref20] Linacre J M. (2019). A User’s Guide to WINSTEPS® Rasch-Model Computer Programs: Program Manual 4.4.6. Mesa-Press, Chicago, IL.

[ref21] Liu J. Cai Y. Lu Q. Song Q. Zhang J. (2022). The influence of involution on the intention of postgraduate entrance examination-based on binary logistic regression model. Eurasia J. Sci. Technol. 4, 43–52.

[ref22] Malec J. F. Torsher L. C. Dunn W. F. Wiegmann D. A. Arnold J. J. Brown D. A.. (2007). The mayo high performance teamwork scale: reliability and validity for evaluating key crew resource management skills. Simul. Healthc. 2, 4–10. doi: 10.1097/SIH.0b013e31802b68ee, PMID: 19088602

[ref23] Mallinson T. Stelmack J. Velozo C. (2004). A comparison of the separation ratio and coefficient α in the creation of minimum item sets. Med. Care 42, I17–I24. doi: 10.1097/01.mlr.0000103522.78233.c3, PMID: 14707752

[ref24] McAlinden C. Khadka J. Paranhos J. d. F. S. Schor P. Pesudovs K. (2012). Psychometric properties of the NEI-RQL-42 questionnaire in keratoconus. Invest. Ophthalmol. Vis. Sci. 53, 7370–7374. doi: 10.1167/iovs.12-9969, PMID: 22997284

[ref25] Mihara R. (2020). Involution: a perspective for understanding Japanese animation’s domestic business in a global context. Japan Forum 32, 102–125. doi: 10.1080/09555803.2018.1442362 E-Emerging Sources Citation Index (ESCI).

[ref26] Pesudovs K. Burr J. M. Harley C. Elliott D. B. (2007). The development, assessment, and selection of questionnaires. Optom. Vis. Sci. 84, 663–674. doi: 10.1097/OPX.0b013e318141fe75 17700331

[ref27] Planinic M. Boone W. J. Susac A. Ivanjek L. (2019). Rasch analysis in physics education research: why measurement matters. Phys. Rev. Phys. Educ. Res. 15:20111. doi: 10.1103/PhysRevPhysEducRes.15.020111

[ref28] Si J. (2022). No other choices but involution: understanding Chinese young academics in the tenure track system. J. High. Educ. Policy Manag. 44, 1–15. doi: 10.1080/1360080X.2022.2115332

[ref29] Smith E. V. J. (2002). Detecting and evaluating the impact of multidimensionality using item fit statistics and principal component analysis of residuals. J. Appl. Meas. 3, 205–231. 12011501

[ref30] Smith E. V. Wakely M. B. De Kruif R. E. L. Swartz C. W. (2003). Optimizing rating scales for self-efficacy (and other) research. Educ. Psychol. Meas. 63, 369–391. doi: 10.1177/0013164403063003002

[ref31] Tennant A. Conaghan P. G. (2007). The Rasch measurement model in rheumatology: what is it and why use it? When should it be applied, and what should one look for in a Rasch paper? Arthritis Care Res. 57, 1358–1362. doi: 10.1002/art.23108, PMID: 18050173

[ref32] Wang W.-C. (2008). Assessment of differential item functioning. J. Appl. Meas. 9, 387–408. 19092232

[ref33] Wilson M. (2004). Constructing Measures: An Item Response Modeling Approach: An Item Response Modeling Approach. Mahwah: Routledge.

[ref34] Wright B. D. Masters G. N. (1982). Rating Scale Analysis: Rasch Measurement. Chicago: MESA Press.

[ref35] Xue L. (2021). Educational Involution during the Social Transition Period BT-Proceedings of the 2021 4th International Conference on Humanities Education and Social Sciences (ICHESS 2021). 674–678.

[ref36] Yan D. Zhang H. Guo S. Zeng W. (2022). Influence of anxiety on university students’ academic involution behavior during COVID-19 pandemic: mediating effect of cognitive closure needs. Front. Psychol. 13:5708. doi: 10.3389/fpsyg.2022.1005708, PMID: 36248474 PMC9558283

[ref37] Yi D. Wu J. Zhang M. Zeng Q. Wang J. Liang J.. (2022). Does involution cause anxiety? An empirical study from Chinese universities. Int. J. Environ. Res. Public Health 19:9826. doi: 10.3390/ijerph19169826, PMID: 36011462 PMC9408648

[ref38] Yu S. Zheng J. Xu Z. Zhang T. (2022). The transformation of parents’ perception of education involution under the background of “double reduction” policy: the mediating role of education anxiety and perception of education equity. Front. Psychol. 13:800039. doi: 10.3389/fpsyg.2022.800039, PMID: 35664177 PMC9161288

[ref39] Zheng W. D. Jing T. T. Cheng R. J. Li X. (2022). An analysis of the influence path of anxiety on the irrational competition behavior of college students in the post epidemic era—the intermediary role of employment values. Adv. Educ. 12, 918–926. doi: 10.12677/AE.2022.124146

