# Supplementary material for: Psychometric evaluation of the academic involution scale for college students in China: An application of Rasch analysis
Source: Front Psychol. 2023 Feb 21;14:1135658. doi: 10.3389/fpsyg.2023.1135658 (PMC9990466; doi:10.3389/fpsyg.2023.1135658)
Supplement: Supplementary file 2 [file Data_Sheet_1.PDF]

Appendix A Academic Involution Scale for College Students (AISCS)

Factors	Items	Strongly Disagree (1)	Disagree (2)	Neutral (3)	Agree (4)	Strongly Agree (5)
Learning	L1. I will attend a tutorial class privately to improve myself so as not to be left behind by others.<br>我会私下报学习辅导班提升自己以免被别人落下。					
	L2. I will follow some knowledge-sharing social media accounts (such as bilibili accounts, microblog accounts, or Xiaohongshu, etc.) to improve myself, so as to avoid being left behind by others.<br>我会在社交媒体（如 B 站、微博、小红书等）关注一些知识分享类账号提升自己，以免被别人落下。					

	L3. In order to get better results, I will consult with the senior students about the relevant knowledge of the courses I have registered (such as the past exam questions, test materials, and teacher's notes.<br>我会向学长打听所修读课程的相关知识（如历年考试真题、考试资料、老师授课重点以及笔记）以便取得更好的成绩。					
	L4. In order to achieve excellent results on the final exam, I will purchase some learning resources (such as PPT templates, reference books, past exam questions, etc.<br>我会购买一些学习资源（如 PPT 模板、参考书、考题等）以便在期末考试中取得优异的成绩。					
	L5. I will often inquire about my classmates' learning situation, and if I feel that the quality of my homework is not as good as theirs, I will modify it to avoid being left behind by others.					

	我会经常打听同学的学习情况，如果感觉自己的作业质量不如他们的，会对作业进行修改，以免被别人落下。					
	L6. I would go to the library on weekends and other breaks so as not to be left behind.<br>我会在周末及其他休息时间去图书馆看书或学习，以免被别人落下。					
	L7. I get up early and come back late to the dormitory every day to study so as not to be left behind.<br>我每天早起晚归去学习，以免被别人落下。					
Activity	A1. I don't like it very much, but I will participate in various competitions so that my comprehensive evaluation results will not be left behind by others.<br>虽然不是很喜欢，但我会参加各种比赛以便让自己的综合测评成绩不被别人落下。					

	A2. Although I don't like it very much, I will join various clubs so that my comprehensive evaluation results will not be left behind by others.<br>虽然不是很喜欢，但我会参加各种社团以便让自己的综合测评成绩不被别人落下。					
	A3. Although I don't like it very much, I will take part in various voluntary activities so that my comprehensive evaluation results will not be left behind by others.<br>虽然不是很喜欢，但我会参加各种志愿活动以便让自己的综合测评成绩不被别人落下。					
	A4. Although I don't like it very much, I will attend various lectures so that my comprehensive evaluation results will not be left behind by others.<br>虽然不是很喜欢，但我会参加各种讲座以便让自己的综合测评成绩不被别人落下。					

	A5. Although I don't like it very much, I will participate in social practice in winter and summer vacation so that my comprehensive evaluation results will not be left behind by others.<br>虽然不是很喜欢，但我会参加寒暑假社会实践以便让自己的综合测评成绩不被别人落下。					
Social relations	SR1. I will actively help my roommates to avoid losing in various evaluations.<br>我会积极帮助宿舍成员，以免在各种评选中落选					
	SR2. I will keep a good relationship with my classmates to avoid losing in various evaluations.<br>我会跟班里同学搞好关系，以免在各种评选中落选。					
	SR3. I will actively interact with teachers and strive to achieve no lower grades than others.					

	我会跟任课老师积极互动，争取平时成绩不比别人低。					
	SR4. I will actively reply to the tutor's comments to avoid being defeated in various evaluations.<br>我会积极回复辅导员的留言，以免在各种评选中落选。					
